# Investigating the dependency of in vitro benchmark concentrations on exposure time in transcriptomics experiments

**DOI:** 10.1016/j.tiv.2023.105761

**Published:** 2024-03

**Authors:** Donatella Carpi, Roman Liska, Julia M. Malinowska, Taina Palosaari, Mounir Bouhifd, Maurice Whelan

**Affiliations:** European Commission, Joint Research Centre (JRC), Ispra, Italy

**Keywords:** Chemical safety, Hazard assessment, In vitro, Toxicology, Point-of-departure, Benchmark concentration, Transcriptomics

## Abstract

There is increasing interest to employ in vitro transcriptomics experiments in toxicological testing, for example to determine a point-of-departure (PoD) for chemical safety assessment. However current practices to derive PoD tend to utilise a single exposure time despite the importance of exposure time on the manifestation of toxicity caused by a chemical. Therefore it is important to investigate both concentration and exposure time to determine how these factors affect biological responses, and as a consequence, the derivation of PoDs. In this study, metabolically competent HepaRG cells were exposed to five known toxicants over a range of concentrations and time points for subsequent gene expression analysis, using a targeted RNA expression assay (TempO-Seq). A non-parametric factor-modelling approach was used to model the collective response of all significant genes, which exploited the interdependence of differentially expressed gene responses. This in turn allowed the determination of an isobenchmark response (isoBMR) curve for each chemical in a reproducible manner. For 2 of the 5 chemicals tested, the PoD was observed to vary by 0.5–1 log-order within the 48-h timeframe of the experiment. The approach and findings presented here clearly demonstrate the need to take both concentration and exposure time into account when designing in vitro toxicogenomics experiments to determine PoD. Doing so also provides a means to use concentration-time-response modelling as a basis to extrapolate a PoD from shorter to longer exposure durations, and to identify chemicals of concern that can cause cumulative effects over time.

## Highlights

• Dependency of benchmark concentrations (BMC) on exposure time was investigated in vitro using HepaRG cells
• A multivariate analysis was used to derive BMC that utilised the complete concentration-exposure transcriptomics dataset
• Results indicate that BMC can vary with exposure time and the degree of this variation is chemical dependant
• Exposure time should be properly considered when designing in vitro studies to determine BMC for chemical safety assessment

## 1 Introduction

Dose-response analysis is a fundamental step in characterising chemical hazard. The process can yield different points of departure (PoD) useful for a variety of purposes, such as estimating the margin-of-safety in a risk assessment or deriving reference values for limiting exposure through risk management measures (EFSA Scientific Committee et al., 2017; US EPA, 2012). Among the approaches employed for PoD derivation, benchmark concentration modelling is frequently used in regulatory toxicology. A benchmark concentration (BMC) is the concentration of a chemical that produces a predefined level of response in a concentration-response experiment. The BMC is usually determined by fitting a suitable mathematical model to concentration-response data and then finding the concentration that corresponds to the chosen benchmark level. Historically, a BMC has also been referred to as a benchmark dose (BMD) since it has typically been derived from experiments using animal models as test systems.

Measuring a BMC in vitro is receiving increasing attention (Krebs et al., 2019; Macko et al., 2021), reflecting the shift in regulatory toxicology to rely more on non-animal approaches (Pistollato et al., 2021). The range of in vitro methods that can be employed to determine BMC is extensive, including assays based on cellular phenotypic biomarkers (Johnson et al., 2014; Paul Friedman et al., 2020) and more recently, on transcriptomics and metabolomics measurements (Harrill et al., 2021; Malinowska et al., 2023; Ramaiahgari et al., 2019; Serra et al., 2020a). Studies to generate in vitro transcriptomics datasets often use different technical approaches which can influence the calculated BMC value (Harrill et al., 2019). To date, the majority of transcriptomics studies employ experimental designs based on a multi-concentration and single time-point exposure protocol (Guo and Mei, 2018). It is well known, however, that the magnitude, rate and type of toxicological response are nearly always dependant to some degree on the time of exposure, regardless of whether the response is measured as an apical effect in an intact organism (Baas et al., 2010) or a change at the molecular level (Aguayo-Orozco et al., 2018; Tennekes and Sánchez-Bayo, 2013). Therefore, it is important to consider both concentration and exposure time in toxicological experiments to understand how these two factors together drive biological response, as well as the determination of a BMC (Serra et al., 2020b). In addition, current in vitro methodologies only cover short exposure scenarios (e.g. several hours to a few days) and thus robust methods are required to extrapolate findings from shorter to longer exposure durations (ECHA, 2012; Macko et al., 2021).

The derivation of a BMC from transcriptomics data poses a significant challenge in that hundreds if not thousands of individual gene responses need to be analysed and combined in some way, to derive a single BMC value that represents the benchmark response of the entire biological system. A variety of approaches for deriving a BMC have been reported that build on different concepts and mathematical analyses. One distinguishing step between the different approaches is how genes are selected for downstream processing and calculation of a BMC. Some techniques use purely statistical criteria for retaining only representative or responsive genes, whilst others employ gene ontologies to select genes that are associated with toxicological pathways of interest. Certain techniques use a combination of both (Nault et al., 2020). Once the set of representative genes has been established, a mathematical model is usually fitted to each gene response for the determination of a gene-specific BMC. These individual BMCs are then combined to produce one BMC value, for example by calculating the median of all the gene-specific BMC values (Farmahin et al., 2017; Webster et al., 2015). However, current approaches to BMC modelling have limitations since they do not consider the interdependence of genes, or how a toxicological response develops over time. Thus there is a need to devise suitable approaches that take gene interdependence and exposure time into account.

The aim of this study was to explore how, and to what extent, the BMC obtained from an in vitro transcriptomics study varied as a function of exposure time. In addition, recognising and exploiting the fact that individual gene expression responses are usually interdependent and thus correlated, an integrated modelling and analysis approach was developed to provide a more appropriate means of deriving a system-level BMC. It is envisioned that the methodology and findings reported here will support and encourage more robust and comprehensive characterisation of in vitro concentration-response to better inform chemical safety assessment.

## 2 Materials and methods

### 2.1 Cell culture

The test system used in this study was the HepaRG cell line, a metabolically competent human hepatic cell line established by the INSERM laboratory (National Institute of Health and Medical Research) at Rennes, France (Anthérieu et al., 2012; Guillouzo and Guguen-Guillouzo, 2008). Undifferentiated HepaRG cells were provided by Biopredic International (Rennes, France, batch number HPR-101056) in cryopreserved vials and were differentiated and cultured as described in Joossens et al. (2019). In brief, cells were maintained in 150 cm^2^ flasks for two weeks in culture medium consisting of Williams' E Medium (Thermo Fisher Scientific, Melegnano, Italy) with 10% fetal bovine serum (HyClone Fetal- Clone III, HyClone), 1% l-glutamine, 1% penicillin/streptomycin, 5 μg/ml bovine insulin and 50 μM hydrocortisone (Sigma, Milan, Italy). After starting to differentiate into hepatocyte- and biliary-like cells, the cells were cultured in differentiation medium, which consisted of maintenance medium plus 1.7% dimethyl sulfoxide (DMSO) (starting with 0.85% DMSO for 1 day) for 2 weeks. Then, the cell culture was selectively trypsinised as to detach the hepatocytes and not the biliary-like cells which are more adherent. The enriched hepatocyte culture was then transferred into clear bottom polystyrene 96-well microplates (5 × 10^4^ cells/well in 100 μl) where they remained for 72 h before undergoing chemical treatment. All the procedures for cell seeding, serial dilution of test items, cell treatment and cell staining were fully automated on robotic liquid handlers (Hamilton Star and Starlet platforms, Hamilton Italia Srl, Agrate Brianza, Italy).

### 2.2 Chemical treatment of the HepaRG cells

Five chemicals were chosen to elicit a transcriptional response in the HepaRG cells: aflatoxin B_1_ (CAS No. 1162-65-8, purity ≥99.2%, Sigma), benzo[*a*]pyrene (CAS No. 50–32-8, purity ≥99.0%, Sigma), cyclosporine A (CAS No. 59865–13-3, purity >99.9%, Sigma), rotenone (CAS No. 83–79-4, purity >95.9%, Sigma) and trichostatin A (CAS No. 58880–19-6, purity >98%, Sigma). These chemicals have already been studied extensively by other investigators and shown to act through specific modes-of-action (Madia et al., 2020). Based on data previously generated in range-finding tests, a dilution series of 8 concentrations with a dilution factor of 2.5 was prepared for each test item. The highest exposure concentration employed was 50 μM, except for rotenone, which was set at 10 μM. To provide some cellular phenotypic data, cell response was measured at 24 h using high content imaging combined with immunofluorescence assays of 3 multiplexed endpoints, namely nucleus morphology, cell membrane integrity and mitochondrial membrane potential. The results are reported in the JRC's Data Catalogue (Carpi et al., 2018).

The transcriptomics study produced 3 biological replicates, where HepaRG cells were split during culturing and seeded on separate 96-well microplates, and 3 technical replicates, where the corresponding samples from each of the split-cultures were included on the same 96-well microplate. Following chemical treatment, microplates were incubated for 2, 6, 12, 24 or 48 h.

In order to avoid confounding effects related to well location, the 96-well microplate layout was changed for each biological replicate. However the layout followed a generic format in that each 96-well microplate included 4 exposure concentrations (out of 8) and 3 technical replicates for each time-point. In addition, 6 vehicle (solvent) control samples were included in each 96-well microplate corresponding to cells incubated in cell media with 0.1% DMSO. This experimental design yielded a total of 1980 individual samples for gene expression analysis i.e. (5 chemicals × 4 concentrations × 3 technical replicates +6 solvent controls) x 2 96-well microplates x 5 time points x 3 biological replicates.

### 2.3 Measurement of gene expression and selection of differentially expressed genes

The gene expression profile was obtained for each sample by targeted RNA-Seq, a next generation sequencing technology applicable for high-throughput workflows (Limonciel et al., 2018). The measurements were carried out by BioSpyder Technologies (https://www.biospyder.com/) using the Templated Oligo assay with Sequencing readout (TempO-Seq). The technology is based on hybridisation and sequencing of highly specific detector oligos. Detector oligos (DOs) were annealed to adjacent sequences of target RNAs in the cell lysate, after which excess oligos were digested with a nuclease, and remaining oligos were ligated and amplified by polymerase chain reaction (PCR) (Yeakley et al., 2017). Each sample was then sequenced resulting in ‘FASTQ’ files, which were then aligned against the TempO-Seq transcriptome using the STAR aligner (https://github.com/alexdobin/STAR). The output of this analysis generated a data matrix for each sample that reported the read counts of 21,111 DO pairs targeting 19,287 human genes.

Pre-processing and quality control of the data were performed in the R environment (R 3.5.3; packages: DESeq2 version 3.7, edgeR version 3.22.5). Genes with barely detectable expression levels were discarded by applying a cut-off filter of 10 mean reads which resulted in a remaining set of 10,270 genes. The expression levels of these genes were normalised as counts per million, and were then log2 transformed. The dataset was further filtered by removing genes with negligible response, as follows. For each chemical, the genes that were retained had a significant differential expression level for at least one concentration at any time point. The significance was defined as an expression (log2)-fold change > |1| when applying the two-sided Williams' trend test.

### 2.4 Concentration-time modelling of differentially expressed genes

After subtracting the expression of the vehicle controls, the concentration-time response of each gene, represented by a matrix of 8 (concentration) x 5 (time point) values, was subjected to mathematical smoothing using a thin-plate spline technique, implemented using the Matlab software package (function ‘tpaps’). This type of smoothing uses ‘nearest neighbour’ data points to average out small spurious ‘jumps’ in gene response associated with very small changes in concentration or time. It was assumed that these jumps represented noise in the data and were likely caused by technical artefacts in the experiment since the actual biological response of interest was expected to vary more gradually as a function of concentration and exposure time.

The next step was to fit a factor model to each of the gene expression datasets obtained for each biological replicate for each chemical. A factor model has the following form (Eq. (1)):

(1) $$y_{g}\left(c,t\right)=\sum_{r=1}^{p}w_{g,r}f_{r}\left(c,t\right)+e_{g}\left(c,t\right)$$

where $y_{g}\left(c,t\right)$ is the modelled response of gene g at concentration c and exposure time t; $\sum_{r=1}^{p}w_{g,r}f_{r}\left(c,t\right)$ is known as the common part, $f_{r}\left(c,t\right)$ are the orthogonal factors; coefficients $w_{g,r}$ are the loadings for gene g for factor *r*; *p* is the number of factors; and $e_{g}\left(c,t\right)$ is an error term. The factor model was fitted by estimating principal components (PC) with the factors $f_{r}\left(c,t\right)$ representing eigenvectors (Ramsay and Silverman, 2005). Once the factors and loadings were computed, the modelled response of any individual gene could be reconstructed by taking the sum of the desired number of factors, each multiplied by the corresponding factor loading for that gene.

The selection of the appropriate number of factors to reconstruct the gene expression depends on the relative size of the estimated eigenvalues of the corresponding covariance matrix. The relative size of the eigenvalue associated with the first principal component represents the percentage of the total variance that lies in the principal component direction, explained by the first factor $f_{1}\left(c,t\right)$. The same is true for the second eigenvalue, and so on. Given that the total variance is a sum of eigenvalues, the appropriate number of factors for satisfactorily reconstructing the gene expression response depends on the desired amount of explained variance of the responses.

### 2.5 Determining BMC and its dependence on time of exposure

A concentration-response experiment typically produces one BMC value per time point. However, when the response of the test system is a function of both concentration and time, described by a response surface rather than a response curve, the BMC is not a single fixed value but varies with exposure time. Thus graphically, the benchmark response (BMR) can be depicted as an isoBMR curve (contour) superimposed on a concentration-time response surface, which connects different concentration-time point combinations that give the same BMR.

**Fig. 1:**
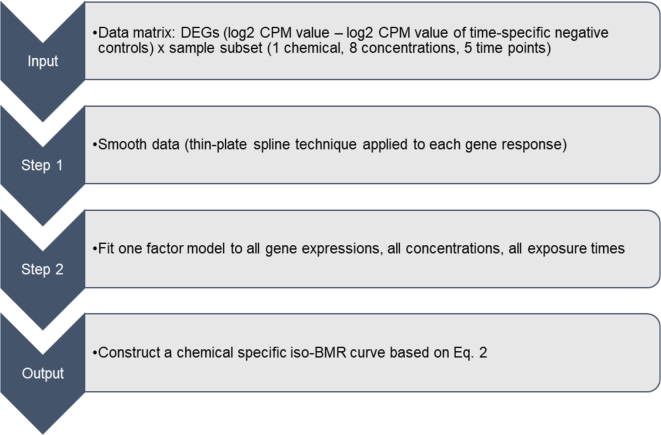
Representation of the workflow applied to determine the isoBMR curves.

The workflow used to obtain isoBMR curves for each chemical is summarised in Fig. 1. First, a one-factor model is fitted to the data (see Eq. (1)) and estimates of $f_{1}\left(c,t\right)$ and loadings$\;w_{g,1}$, denoted by $\hat{f}\left(c,t\right)$ and$\;{\hat{w}}_{g}$ respectively, are obtained.

The median isoBMR curve is then defined as a set of $\left(c,t\right)$ values that solve $\hat{f}\left(c,t\right)={\hat{v}}_{\mathrm{med}}\;$where,

(2) $${\hat{v}}_{\mathrm{med}}={\text{median}}_{g}\left(\frac{1.349\times \sigma_{g}}{{\hat{|w}}_{g}|}\right)$$

and $\sigma_{g}$ is the averaged standard deviation over all exposure times for gene *g*. In the equation the BMR factor (i.e. the number of standard deviations at which the BMD is defined) corresponds to the commonly used value of 1.349. It is a user input used to define the BMR. IsoBMR curves can be derived applying other BMR factors. Percentiles other than median can be obtained in a similar way.

The median isoBMR curve is representative of all gene specific isoBMR curves combined. However, any gene specific isoBMR curve can be generated from the set of $\left(c,t\right)$ values that solve the equation (Eq. (3)),

(3) $$\hat{f}\left(c,t\right)=\frac{1.349\times \sigma_{g}}{\left|{\hat{w}}_{g}\right|}$$

The gene specific isoBMR curves (for a given chemical) are parallel to each other since their associated response surfaces are all derived from a rescaling of the common underlying factor $\hat{f}\left(c,t\right)$.

## 3 Results

### 3.1 Number of differentially expressed genes responding to chemical exposure

The number of differentially expressed genes aggregated over all concentrations, for each time point and chemical, are shown in Table 1. For all chemicals, the number of differentially expressed genes at the earliest time point (2 h) was <5% of the corresponding total. For aflatoxin B_1_, rotenone, and trichostatin A, the number of differentially expressed genes increased over the exposure time of 48 h. For cyclosporine A, this number increased over 24 h and then slightly decreased at 48 h, whilst for benzo[*a*]pyrene a small fluctuation was observed within the 2 to 6 h interval. In addition, for aflatoxin B_1_, trichostatin A, and benzo[*a*]pyrene, the number of differentially expressed genes exceeded 30% of the total for the final time point, suggesting a significant perturbation of normal cellular function (Bourdon-Lacombe et al., 2015).

**Table 1:**
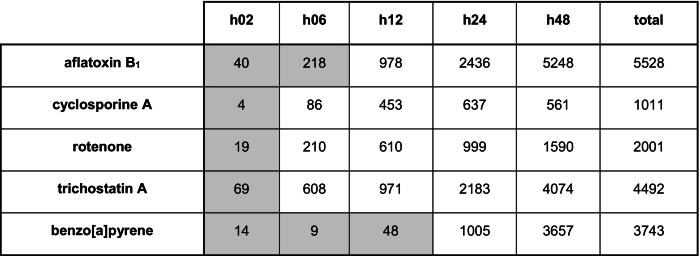
The total number of differentially expressed genes (Williams' test *p*-value <0.05, log2 fold change >1) combined for all concentrations, corresponding to each time point and chemical. The last column reports the total number of unique differentially expressed genes across all concentrations and time points for each chemical. The grey-shaded cells indicate where the number of differentially expressed genes is <5% of the total number of genes measured.

### 3.2 Factor modelling of differentially expressed genes

**Fig. 2:**
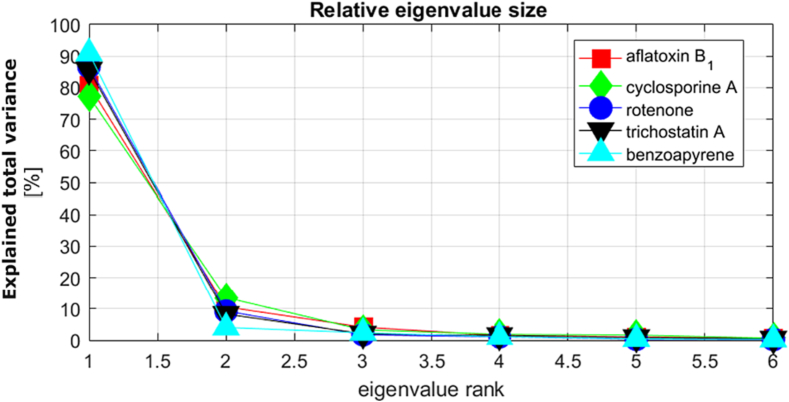
Relative magnitude of eigenvalues for each chemical dataset corresponding to the covariance matrices of differentially expressed genes averaged over biological replicates.

To investigate the degree of interdependence between differentially expressed genes, relative eigenvalue magnitudes were plotted for each chemical dataset (Fig. 2). The relative size of the first eigenvalue for each chemical varied from 77% to 91%, decreasing to 4–14% for the second eigenvalue, and to very low levels thereafter. This finding underpinned the choice of using a one-factor model to determine BMC as described earlier (Section 2.5).

**Fig. 3:**
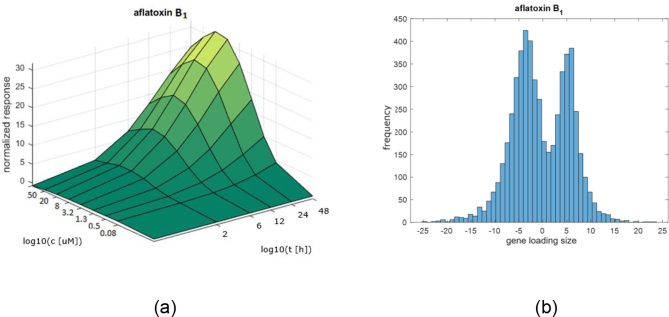
Results derived from an aflatoxin B_1_ dataset: a) an example of a shape of a response surface $\hat{f}\left(c,t\right)$, b) an example of a histogram of a distribution of gene loadings ${\hat{w}}_{g}$.

The typical shape of the common underlying factor response $\hat{f}\left(c,t\right)$ for aflatoxin B_1_ is illustrated in Fig. 3a. An example of a distribution of gene loadings calculated for an aflatoxin B_1_ dataset is shown in Fig. 3b.

When the common factor surface $\hat{f}\left(c,t\right)$ is non-negative for all values of $\left(c,t\right)$ then the sign of a gene loading ${\hat{w}}_{g}$ indicates whether the (fitted) response for that gene is up- or down-regulated.

### 3.3 IsoBMR curves

**Fig. 4:**
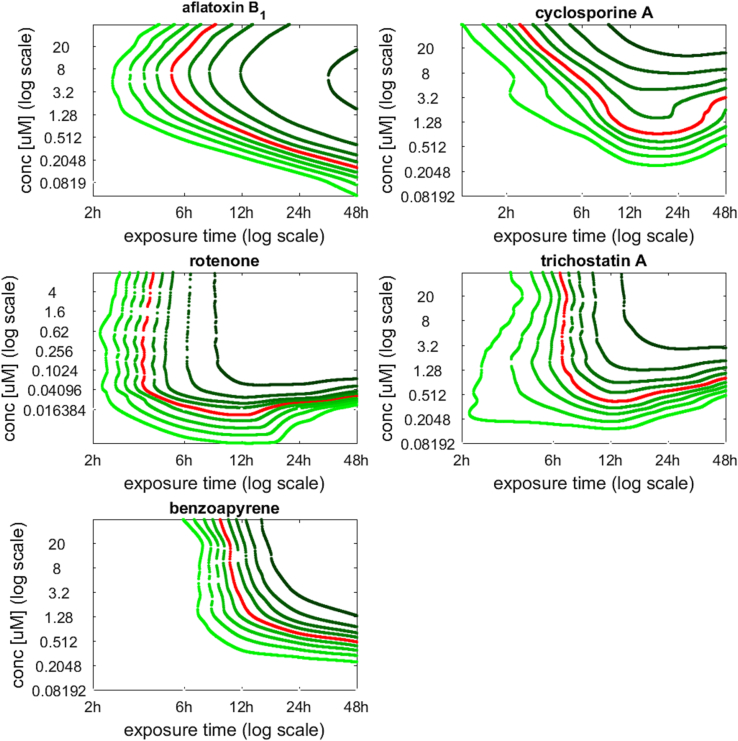
Percentile isoBMR curves for the five chemical datasets. The isolines were retrieved for the 10th, 20th, etc., percentiles. The red line was obtained at the 50th percentile corresponding to the median response. (For interpretation of the references to colour in this figure legend, the reader is referred to the web version of this article.)

The isoBMR curves for each chemical, for the averaged response of the 3 biological replicates, are shown in Fig. 4. Iso-BMR curves are reported in log-log scale, according to a classical approach to visualise how the toxicological effect of a chemical changes across time and concentration/dose (Miller et al., 2000). The isoBMR curves may be interpreted as follows, taking the 10th percentile as an example: the isoBMR <10th percentile> curve determines the border where 10% of the genes have isoBMR curves which are more ‘sensitive’ i.e. shifted down and left, which can also be interpreted as these genes responding earlier to chemical treatment. The red line depicts the median isoBMR curve where half of the genes have isoBMR curves that are more sensitive, and half which are less sensitive. Taking any isoBMR curve, through projection, it is possible to obtain the BMC which corresponds to a particular exposure time of interest.

**Fig. 5:**
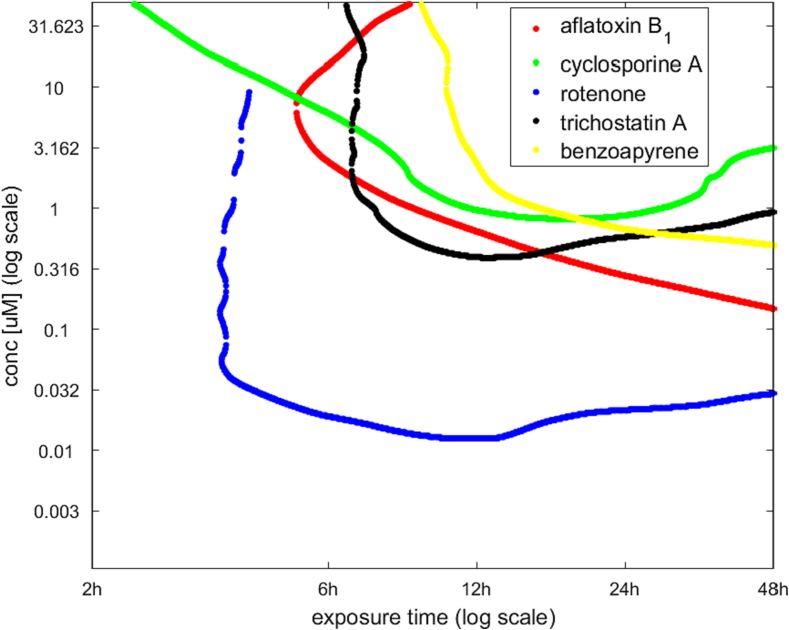
Median isoBMR curves for the five chemicals obtained from in vitro transcriptomics data. The curves depict the average response of three biological replicates.

Co-plotting the isoBMR curves for all chemicals tested in the study allowed a straightforward comparison with respect to their apparent potency in this particular in vitro assay (Fig. 5). For example, it was evident that the lowest concentrations of rotenone affected gene expression in the HepaRG test system, whilst cyclosporine A generally appeared to be the least potent. With respect to benzo[*a*]pyrene, this chemical induced its toxic effect over longer exposure times (i.e. 12 h onwards). The slope of the isoBMR curves can provide information on the dynamic characteristic of these chemicals where for example, a steep downward slope may indicate a cumulative toxicological effect with increasing time of exposure. For rotenone, cyclosporine A and trichostatin A, excluding their very early transient responses, their BMC remained more or less the same over the exposure period, whilst for benzo[*a*]pyrene and aflatoxin B_1_, their BMC decreased with increasing exposure time (Fig. 5).

**Fig. 6:**
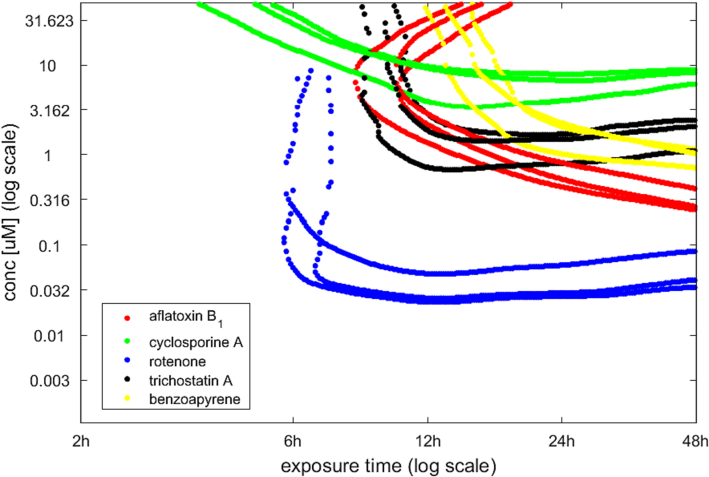
Median isoBMR curves for the five chemicals obtained from in vitro transcriptomics data. The curves depict three biological replicates individually allowing to investigate the reproducibility of the entire in vitro transcriptomics workflow.

Lastly, to evaluate experimental reproducibility, the median isoBMR curves were plotted separately for each biological replicate and chemical, as shown in Fig. 6. The shape and slope of each biological replicate per chemical were consistent, indicating satisfactory reproducibility of the experiment itself and the transcriptomics analysis workflow. It should be noted however that for all the chemicals, the isoBMR curve associated with one biological replicate was slightly offset from its two corresponding replicates.

## 4 Discussion

The integrated multivariate data analysis approach based on factor modelling proved to be convenient and robust to model the collective response of all differentially expressed genes. The notable interdependence of gene responses was evidenced through the eigenvalue analysis carried out (Fig. 2) and explains the fact that a simple one-factor model was able to describe a significant amount of the variation (77% to 91%) of all gene responses. This in turn provided a straightforward approach of determining an isoBMR curve for each chemical, based on a percentile of gene loadings chosen as being representative of the total system response.

Two chemicals tested in this study (benzo[*a*]pyrene and aflatoxin B_1_) showed a notable dependency of in vitro BMC on exposure time (Fig. 4, Fig. 5 and Fig. 6). The reduction of BMC over time was approximately between 0.5 and 1 log-order in magnitude, which is appreciable considering that the maximum exposure time was only 48 h. The reason for this observation is likely related to mode-of-action, which in the case of these two chemicals involves metabolic biotransformation to more toxic products (Bukowska et al., 2022; Rushing and Selim, 2019) that is influenced by metabolic competence of the HepaRG test system (Vlach et al., 2019). On a practical level, these findings indicate that the choice of exposure time in an in vitro transcriptomics study to determine BMC is an important factor to consider. For example, it could be taken into account through a suitable experimental design or when characterising uncertainties associated with reported BMC values.

The observed dependency of BMC on exposure time for some chemicals in this study may be indicative of the same phenomenon that is often observed in more conventional toxicological tests (Rozman and Doull, 2001; Rozman and Doull, 2000). In general terms, the dependency of the magnitude of a certain toxicological effect on both concentration (dose) and exposure time is often described by Haber's rule (i.e. *c* x *t = k*, where *c*, *t* and *k* denote concentration, time and effect, respectively). In an experiment that follows this rule, doubling the exposure time requires half of the concentration to achieve the same level of effect, and vice versa. However, the relationship between exposure concentration, time and effect can deviate from Haber's rule, in which case a more general power law usually proves a better fit (Miller et al., 2000). Knowing the actual relationship between concentration, exposure time and effect is important for the hazard characterisation of a chemical since it provides a good basis to extrapolate a PoD from shorter to longer duration time points, avoiding the use of default methods (e.g. that assume Haber's rule to apply) that may underestimate or overestimate the actual hazard (Miller et al., 2000). Measuring and modelling the relationship between concentration, exposure time and effect also provides important insights into the dynamic nature of a chemical's mode-of-action. For example, a chronicity index can be determined to provide a quantitative measure of a chemical's potential to cause cumulative effects over time (Macko et al., 2021), which is an important consideration in hazard assessment and the setting of safe exposure limits. With this in mind therefore, the results presented here (Fig. 4 and Fig. 5) could be interpreted as indicating a cumulative toxicological mode-of-action for two of the chemicals tested (benzo[*a*]pyrene and aflatoxin B_1_). For the others, the exposure time appeared to have little effect and thus the gene response was primarily a function of concentration, which in pharmacological terms would be equivalent to a ‘C-max’ scenario (Macko et al., 2021).

The discussion above about the relationship between concentration, exposure time and toxicological effect raises a more fundamental question about the nature of system response at the level of gene transcription, when compared to responses at higher levels of biological organisation. Most of the knowledge gained over the last 100 years or so, to understand the complex and context-specific relationship between concentration, exposure time and toxicological response, has been derived from empirical observations made in whole organism studies, many using death as the measure of effect. More recently, in vitro studies of cell viability as a function of concentration and time of exposure indicate that general Haber-like behaviour at organism level apparently translates to the cellular level (Macko et al., 2021). Assuming this is the case, then modelling and extrapolation approaches originally designed for in vivo studies can be simply repurposed and applied to in vitro methods with a cellular (phenotypic) endpoint. However, when measuring system response at the molecular level using transcriptomics or metabolomics for example, under what conditions is it reasonable to assume that Haber-like behaviour still holds? On one hand, advanced mathematical modelling of molecular networks (Ay and Arnosti, 2011) highlight the huge complexity and significant nonlinearity of molecular-level dynamics, hinting, in simplistic terms, that it is not necessarily reasonable to expect Haber-like response at the molecular level. On the other hand, the results described here and in several other studies do not seem to indicate anything very different from Haber-like behaviour. One reason for this, in this study, might be the fact that the overall response of the system was determined from the collective response of all differentially expressed genes, which may have suppressed some ‘unusual’ dynamics of a subset of genes. This deserves further consideration and investigation.

## 5 Conclusions

This study has shown that the PoD derived from an in vitro concentration-response transcriptomics experiment can vary significantly with exposure time, even in typical short-term assays. This finding generally corresponds to what has been extensively observed over a hundred years or more in the toxicological testing of chemicals in whole organisms. Taking this fact into consideration when designing in vitro transcriptomics studies will not only help to avoid misleading PoD results, but will also provide important information on mode-of-action dynamics that supports the identification of substances of concern, and the extrapolation of PoD values for the determination of safe exposure levels in chronic exposure scenarios. Another prominent finding in this study was the high degree of interdependency in differential gene expression, which indicated that although individual genes may be up- or down-regulated with different magnitudes, the majority of gene-responses tended to move in unison. On a practical level, this allowed the use of a convenient multivariate data analysis approach based on factor-modelling to describe the collective response of all differentially expressed genes with respect to both concentration and exposure time, and to determine isoBMR curves for each of the chemicals tested. On a more fundamental level, this suggests that optimally designed concentration-time response experiments combined with non-parametric multivariate modelling may prove an efficient and effective means of both filtering and grouping genes and categorising chemicals according to modes-of-action described by response dynamics.

## Declaration of Competing Interest

The authors declare that they have no known competing financial interests or personal relationships that could have appeared to influence the work reported in this paper.

## Data Availability

As reported in the manuscript, data presented in this study are available in the public repository BioStudies with reference E-MTAB-13168.
